# Flexible Phenology of a C_4_ Grass Linked to Resiliency to Seasonal and Multiyear Drought Events in the American Southwest

**DOI:** 10.1002/ece3.71435

**Published:** 2025-05-14

**Authors:** Rebecca A. Finger Higgens, David L. Hoover, Anna C. Knight, Daniel R. Schlaepfer, Michael C. Duniway

**Affiliations:** ^1^ US Geological Survey Southwest Biological Science Center Moab Utah USA; ^2^ USDA‐ARS Rangeland Resources and Systems Research Unit Fort Collins Colorado USA; ^3^ US Geological Survey Southwest Biological Science Center Flagstaff Arizona USA; ^4^ Center for Adaptable Western Landscapes, Northern Arizona University Flagstaff Arizona USA

**Keywords:** C_3_ grass, C_4_ grass, drought, monsoon, phenology, resiliency, seasonal

## Abstract

Rising temperatures are predicted to further limit dryland water availability as droughts become more intense and frequent and seasonal precipitation patterns shift. Vegetation drought stress may increase mortality and cause declines and delays in phenological events, thereby impacting species' capacity to persist and recover from extreme drought conditions. We compare phenological responses of two common dryland perennial grass species, *Achnatherum hymenoides* (C_3_) and *Pleuraphis jamesii* (C_4_), to 4 years of experimentally imposed precipitation drought treatments (cool season, warm season, ambient), followed by 2 years of recovery on the Colorado Plateau, United States of America. Tagged individual grasses from both species were monitored biweekly and assessed for phenological metrics and mortality. The C_3_ grass exhibited less phenological flexibility to both seasonal and interannual drought conditions and experienced high rates of mortality, thus reducing resiliency. Conversely, the C_4_ grass showed more phenological plasticity during imposed drought treatments, with treatment effects diminishing in the two‐year recovery period during a severe ambient drought. Synthesis: Results suggest that plant photosynthetic strategies may impact plant resistance and resiliency to drought. Here, C_3_ grass populations may decline, potentially shifting cool dryland ecosystems into a system comprised predominantly of warm‐season adapted species.

## Graphical abstract

This study examines how two perennial grass species in the Colorado Plateau respond to seasonal drought conditions and subsequent recovery. The C_3_ grass (*Achnatherum hymenoides*) exhibited lower phenological flexibility and higher mortality, while the C_4_ grass (*Pleuraphis jamesii*) demonstrated greater resilience and recovery despite drought stress. Findings suggest that differing photosynthetic strategies influence drought resistance, potentially leading to shifts in dryland ecosystems toward warm‐season species dominance.

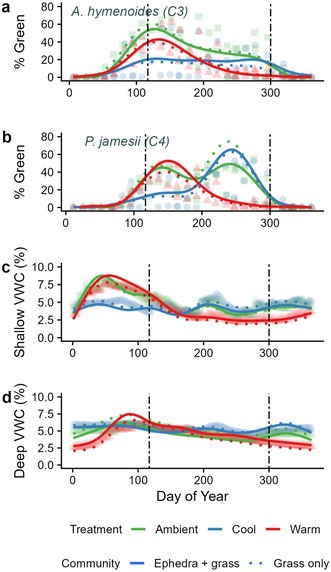

## 1 Introduction

Ongoing climate change is impacting the hydrological cycle, leading to shifts in the seasonal timing of precipitation and increased frequency and intensity of droughts (Williams et al. 2020; Zhang et al. 2022; Swain et al. 2025). Water‐limited dryland ecosystems are predicted to be particularly sensitive to deviations in climate at both annual and seasonal time scales (Brown et al. 1997; Gremer et al. 2018; Zhang et al. 2024). Soil moisture serves as a key regulator of vegetation function and structure in drylands by controlling photosynthesis, biomass production, reproductive activities, and plant mortality (Munson et al. 2011; Moore et al. 2015; Berdugo et al. 2020; Bradford et al. 2020). Current climate projections for many drylands trend toward increased aridity with more frequent droughts, both within and between seasons, which might decrease vegetation cover (Reynolds et al. 2007; Bradford et al. 2020; Smith et al. 2024). To better understand how dryland ecosystems may respond to the seasonal timing of drought, it is important to consider why and how different vegetation community members might respond to shifts in soil moisture availability at both intra‐ and interannual scales.

For many dryland plant species, resistance and resiliency to drought conditions can depend on plant functional traits and adaptations (Tilman and Downing 1994; Volaire 2018; Hoover et al. 2021). Drought resistance occurs when a species can maintain basic biological activity during periods of low moisture without large declines in biological function, while drought resiliency can be measured by how completely and quickly a species recovers once a drought event has subsided (Tilman and Downing 1994; Hoover et al. 2021). Drought‐tolerant plant species will respond to dehydration by rapidly decreasing stomatal conductance to limit tissue water deficits and structural damage; however, this strategy limits carbon uptake and photosynthesis (Kramp et al. 2022). Plants can also adopt a drought escape strategy, which allows plants to complete or shorten the life or reproductive cycle before the onset of drought (Volaire 2018). Conversely, drought‐tolerant plants will maintain relatively high stomatal conductance to maintain carbon assimilation even during periods of low soil water availability, which in severe circumstances can result in plant mortality due to embolism and cavitation in plant vascular structures (McDowell et al. 2008; Skelton et al. 2015). This could mean that drought‐tolerant species may be more vulnerable to mortality events than drought‐avoidant species, which would directly impact drought resilience. However, drought‐induced mortality can be difficult to predict and requires further study (McDowell et al. 2008; Winkler et al. 2019).

Photosynthetic pathways (C_3_ vs. C_4_) are known to contribute to drought survival strategies, with C_4_ photosynthetic pathways optimized for higher temperatures and with greater water‐use efficiency when compared to C_3_ species (Ehleringer et al. 1997; Sage and Kubien 2007; Havrilla et al. 2022). However, C_4_ photosynthesis is more energetically expensive than C_3_ photosynthesis, so C_4_ species have historically only dominated in hotter, low‐latitude environments, while C_3_ species dominate in cooler habitats and/or earlier periods in the annual growing season (Sage and Kubien 2007; Havrilla et al. 2022). If drought events become more intense, frequent, or increase in duration, C_4_ photosynthetic pathways may prove to be more advantageous, as these more conservative strategies may decrease mortality events. In this scenario, ecosystems that are currently mixed with C_3_ and C_4_ plants may see a shift in community composition toward more C_4_ dominance as water limitations intensify (Munson et al. 2011; Witwicki et al. 2016; Havrilla et al. 2022). Conversely, research in other mixed C_3_/C_4_ grasslands has shown that C_3_ grasses can expand in periods of low rainfall and high temperatures when there is a strong shift in seasonal patterns of precipitation, with declines of warm season rains and concurrent slight increases in cool‐season precipitation (Knapp et al. 2020). Therefore, it is important to consider not only the intensity (i.e., percentage reduction in precipitation) but also the timing and seasonality of drought events when projecting shifts in C_3_/C_4_ grass dynamics (Winslow et al. 2003).

Plant phenology, the timing of annual life history events, is known to be sensitive to changes in climate and could be an important indicator of a plant's adaptive strategies to withstand drought. In many more mesic systems such as temperate and tropical ecosystems, global syntheses suggest that warming temperatures can lead to earlier spring green‐up (Piao et al. 2019; Vitasse et al. 2022; Wolkovich et al. 2022). However, in drylands where water is often available only during short pulses, seasonal moisture dynamics may play a more important role in the growth patterns of perennial vegetation (Moore et al. 2015; Wertin et al. 2017; Castillioni et al. 2022; Currier and Sala 2022; Warter et al. 2023). Reductions in seasonal or annual precipitation or warming‐induced soil desiccation are predicted to shorten the length of the grass‐growing season, due both to delays in the timing of green‐up and an earlier onset of senescence (León‐Sánchez et al. 2020; Lu et al. 2023). Grass species sensitivity to seasonal moisture availability or deficit will vary with photosynthetic pathway, with C_3_ grasses relying on elevated moisture and cooler temperatures to stimulate green‐up during spring or fall (Comstock and Ehleringer 1992; Winslow et al. 2003). C_4_ grasses are often considered to be more opportunistic of soil water availability as they can complete all phenological stages during cooler shoulder seasons (i.e., early spring and late fall) as well as during hot summer precipitation events (Comstock and Ehleringer 1992; Schwinning et al. 2008; Castillioni et al. 2022). Together, this indicates that C_3_ grasses may be more reliant on cool‐season precipitation and therefore more sensitive to cool‐season drought than C_4_ grasses, which are better able to utilize warm‐season precipitation.

To address individual grass species responses to seasonal drought conditions, we imposed a 4‐year experimental drought (2015–2019), followed by 2 years of recovery (2019–2021) in a mixed grassland on the Colorado Plateau (Hoover et al. 2021; Finger‐Higgens, Hoover, et al. 2024). The Colorado (CO) Plateau is in the southwestern United States (US), with cool winters, hot summers, and a mean annual temperature of 12.5°C, and relatively low annual precipitation (mean annual precipitation ~220 mm). Monthly precipitation is similar year‐round, with winter moisture originating from winter storm fronts from the Gulf of Alaska, and summer convective and monsoonal storms from the Gulf of California (Hereford and Webb 1992; Schwinning et al. 2008). However, soil water availability varies greatly due to seasonal differences in temperature, evaporative demand, and plant water use (Comstock and Ehleringer 1992; Gremer et al. 2015; Chenoweth et al. 2023). Grasslands and shrublands dominate mid‐elevation sites on the Colorado Plateau, with both C_3_ and C_4_ dominant grasses and shrubs. Additionally, the Colorado Plateau is facing a multidecadal megadrought, punctuated by years of more severe drought conditions (Williams et al. 2020, 2022; Mankin et al. 2021; Finger‐Higgens et al. 2023). This creates an urgent need to further understand how individual plants may respond to different drought conditions and what that could mean for future vegetation distributions, especially in this vulnerable and rapidly changing locale in the American Southwest.

Here we examined how dominant grasses with different photosynthetic pathways (C_3_ vs. C_4_) respond to changes in seasonal moisture, as observed through shifts in phenology and mortality rates both during and after a seasonal drought experiment. We hypothesized that C_3_ grasses would be more sensitive (i.e., delayed phenology and shorter growing seasons) than C_4_ grasses when winter and spring soil moisture was reduced. C_4_ grasses would experience shorter growing seasons in response to drier summer soil conditions. Additionally, we hypothesized that the C_3_ grasses would have a greater rate of mortality in response to drought conditions than C_4_ grasses, due to plant physiological trade‐offs in photosynthetic pathways. We also predicted that grasses grown near large shrubs ( *Ephedra viridis* ; Mormon Tea) would have lower survival rates and further reductions in growing season length due to increased competition for soil water. We hypothesized that competition with *E. viridis* would impact focal grass species the most when winter/spring soil moisture is limited due to the competitive advantages of a deeper‐rooted, evergreen shrub that can access deep soil moisture before grasses green up/emerge. Additionally, we predicted that *P. jamesii* would show faster recovery following the conclusion of the drought experiment, due to the competitive physiological advantages of C_4_ pathway photosynthesis under hot temperatures and drier conditions.

## 2 Materials and Methods

This study was conducted in a mixed shrub grassland ecosystem on the Colorado Plateau in southeastern Utah, US, near the Needles District of Canyonlands National Park (38.19′N, 109.75′W; 1505 m elevation; Hoover et al. 2021, Finger‐Higgens, Hoover, et al. 2024). We focused on the phenology and mortality of two dominant perennial grass species, Indian Ricegrass ( *Achnatherum hymenoides* , C_3_ bunchgrass) and James' galleta grass ( *Pleuraphis jamesii* ; C_4_ rhizomatous grass). Additionally, we tested for the effects of potential competition with the dominant C_3_ evergreen shrub, Mormon tea ( *Ephedra viridis* ) in half of the plots. This allowed us to establish two different community types based on the presence or absence of the shrub *E. viridis* in one half of each plot (grass‐only community and grass with *E. viridis* community; as described in Hoover et al. 2021). Prior to site establishment in 2015, the area experienced intermittent cattle grazing, but a 3.2 ha fenced cattle exclosure was installed prior to the initiation of this study. Soils in the area are very deep (> 1.5 m) loamy fine sands (Finger‐Higgens, Knight, et al. 2024). Within the fenced area, six replicate blocks were established, and each block contained one replicate of each drought treatment and community combination, for a total of 36 plots (Hoover et al. 2021).

Long‐term climate records (1966 to 2021) of daily precipitation sums and maximum and minimum temperatures, were obtained from the visitor center for Canyonlands National Park Needles District (located approximately 3 km from study site and at a similar elevation), through the Western Regional Climate Center (http://www.wrcc.dri.edu). Across the historical data collection period, we calculated daily mean temperature and daily mean precipitation to assess how environmental conditions during our study period deviated from long‐term climate conditions (Grote et al. 2023).

### 2.1 Seasonal Drought Treatments

From May 2015 to April 2019, three precipitation manipulation treatments were designated, including: a control (ambient precipitation), cool‐season drought (66% ambient precipitation excluded approximately November through April), and warm season drought (66% ambient precipitation excluded approximately May through October) (Hoover et al. 2021). Cool‐season rainout shelters were in place from late October to late April (moved between day of year 115–117), while warm season drought plots had shelters in place from late October/early November to late April (moved between day of year 292–310). All shelters were finally removed at the beginning of April 2019 for a total of 4 cycles of both drought treatments. Hourly air temperature and precipitation were also measured on site (Grote et al. 2023). Additionally, soil moisture probes were installed in each plot, at shallow (0–25 cm) and deep (30–50 cm) integrated soil moisture profiles (Hoover et al. 2021; Grote et al. 2023; Finger‐Higgens, Hoover, et al. 2024).

### 2.2 Phenology and Mortality

From 2016 to 2021 we measured plant phenology every 2 weeks on five individuals per plot of each of the two target grass species (*A. hymenoides and P. jamesii*) as described in Hoover et al. (2021). During biweekly phenology sampling, tagged individuals were assessed for greenness (visually estimated as the percent of standing biomass that was green, to the nearest 5%) and phenologic stage. When possible, the same researcher conducted visual estimates of plant greenness to minimize bias and offsets. Phenological metrics were then calculated for each tagged individual and year: (a) start of growing season (SOS), defined as the earliest day of the year on which the individual was not dormant (greenness > 0%); (b) end of growing season (EGS), defined as the last day of the year on which the individual was not dormant (i.e., green tissues were still present); (c) growing season length (GSL), defined as the number of days between the start and end of the growing season for each individual; (d) start of reproductive activity (SRA), defined as the first observation of a mature inflorescence; (e) end of reproductive activity (ERA), defined as the last observation of a mature inflorescence; (f) reproductive activity length (RAL), calculated as the length of time from first flowering to when all inflorescences had senesced (Table 1) (Hoover et al. 2021). Additionally, tagged individuals allowed us to track rates of mortality, where an individual was declared dead if no green leaves appeared in a growing season and all subsequent growing seasons.

**TABLE 1:** Phenology metrics measured for *Achnatherum hymenoides* (C_3_ grass) and *Pleuraphis jamesii* (C_4_ grass).

Abbreviation	Definition
SOS	Start of Growing Season
EGS	End of Growing Season
SRA	Start of Reproductive Activity
ERA	End of Reproductive Activity
GSL	Growing Season Length (EGS‐SOS)
RAL	Reproductive Activity Length (ERA‐SRA)

### 2.3 Statistical Analyses

Precipitation and temperature data from the site's weather station during the study period were compared to long‐term climate data (1966–2016) collected from the nearby Canyonlands National Park Needles District Visitor Center weather station. Temperature deviations from the mean were calculated as the average daily deviation from the 51‐year average in 5‐day increments, fit with a generalized additive model (GAM). GAMs are nonparametric generalized regression models that fit smoothing curves using cubic splines and allow for random effects and autoregressive variance structures (Pedersen et al. 2019). Temperature anomaly was calculated as the 6‐month rolling mean temperature deviation from the 51‐year average temperatures for that window. Relative precipitation of the three precipitation treatments (i.e., ambient, cool‐season drought, and warm season drought) was calculated as the percentage of accumulated precipitation in the preceding 6 months compared to the 51‐year average total precipitation for that window (sensu Fick et al. 2016).

To explore the potential differences between *A. hymenoides* and *P. jamesii* phenology and soil moisture conditions across treatments and plant community types during the experimental period (2016–2018) in shallow (5–25 cm) and deep (30–50 cm) depths, we constructed GAMs. Here, our GAMs used plot ID with nested plant ID as a random effect, and year was used for autoregressive variance structure to account for repeated measures.

Phenology data were standardized prior to analysis to account for individuals that were dying or dead. This was done by standardizing percent greenness per individual plant per year by the maximum greenness observed in that year and excluding individuals that did not appear green that year. Phenology metrics SOS, EGS, SRA, ERA, GSL, and RSL were analyzed using linear mixed effects models. Initial models were constructed with fixed effects of year, drought treatment, and plant community, with a random effect of block with nested plot ID and plant ID, but plant community was ultimately dropped from the models as it was not a significant predictor (as determined by the lowest Akaike Information Criteria (AIC) value through backward step regression (Bates et al. 2015)). For SOS, a log transformation was used to meet model assumptions of normality and homoscedasticity of residuals.

For plant mortality, initially we fit a binomial GAM (non‐linear trend) for both *A. hymenoides* and *P. jamesii* with treatment and plant community as a fixed effects and a smoothing term of year × treatment, but plant community was again not significant and dropped from the final models. However, for *P. jamesii*, the fitted smoothing terms' effective degrees of freedom (EDF) were not significant and showed a linear pattern (EDF = 1) so we fit a binomial generalized linear model (linear trend) for *P. jamesii* with parametric variables of year and treatment. Treatment was ultimately removed from the final *P. jamesii* model as it was not a significant predictor and did not improve model performance as determined by AIC.

All statistical analyses were conducted in R (version 4.4.0) (R Core Team 2022) and data used in this study are published in Finger‐Higgens et al. (2025).

## 3 Results

### 3.1 Environmental Variation During Study Period

**FIGURE 1:**
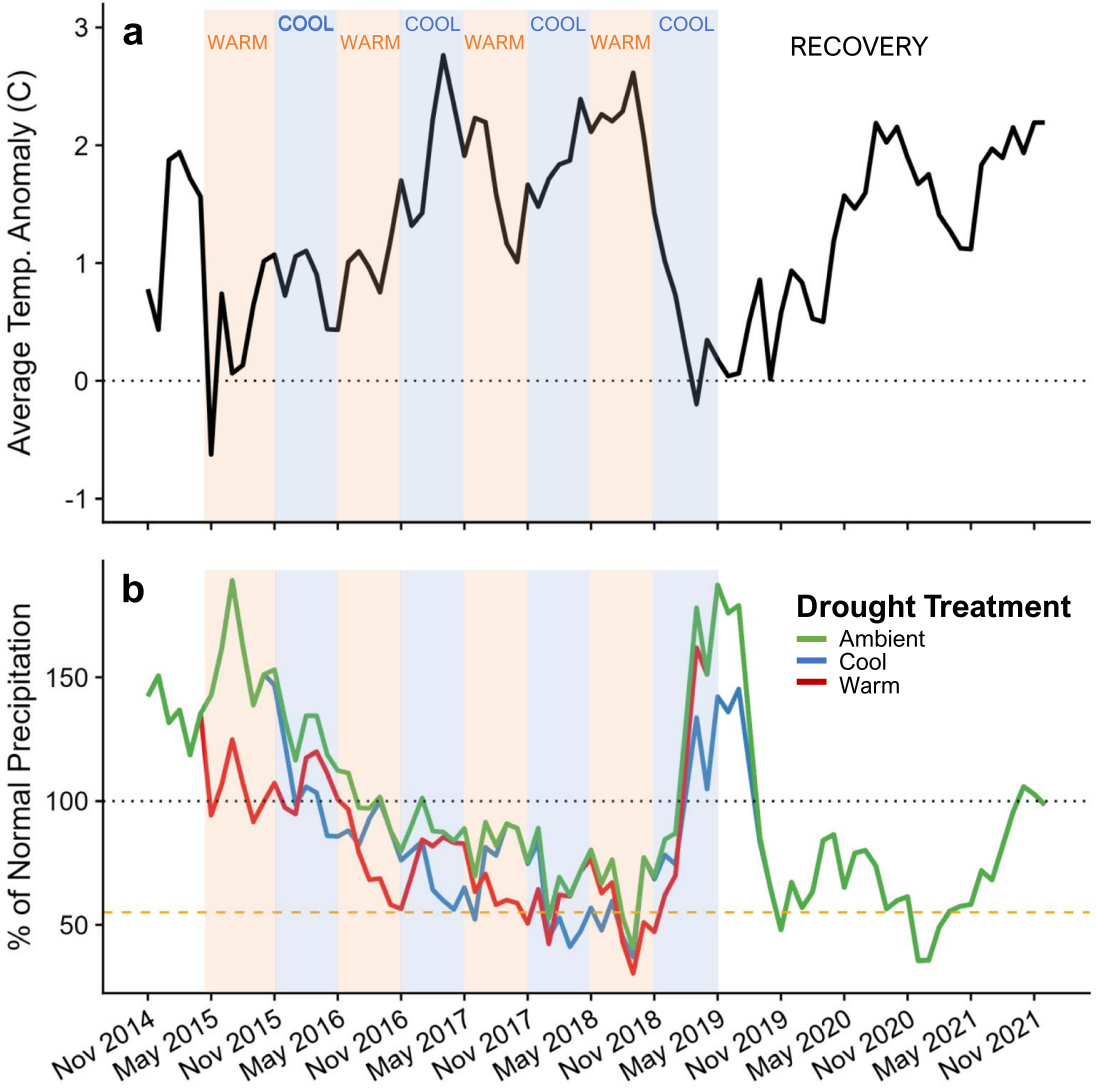
Climate conditions during the experiment (April 2015–April 2019) and subsequent recovery years (April 2019–Dec. 2021) compared to daily deviation from the 50‐year average (calculated from weather data from the Canyonlands National Park Needles District Visitor Center weather station). Temperature anomaly (a) was calculated as the six‐month rolling mean temperature deviation from the 50‐year average temperatures for that window. Relative precipitation (b) of the three drought treatments, ambient (green lines), cool‐season drought (blue lines), and warm season drought (red lines), calculated as the percentage of accumulated precipitation in the preceding 6 months compared to the 50‐year average total precipitation for that window. Red (warm) and blue (cool) shading from May 2015 to May 2019 represent active drought treatment for that time segment. Drought treatment precipitation was calculated from the single weather station placed in situ with associated reduction for periods when shelters were in place.

Over the duration of this study from 2015 to 2021, mean daily temperatures were 1.32°C above the 1966–2016 average conditions (Figure 1a). Precipitation anomalies varied both intra‐ and inter‐annually over this time, with accumulating impacts on the different treatments. During three out of the four years of the experiment, ambient precipitation totals were below the 51‐year average. The only period/treatment combinations with above average precipitation were from April 2015 to April 2016 for ambient and cool drought plots (and intermittently for warm drought plots), and from Oct. 2018 to May 2019 for all treatments (Figure 1b). Throughout most of the experimental years of the study, the warm drought treatments received the least amount of annual precipitation (2016: 164 mm, 2017: 130 mm, 2018: 137 mm) followed by the cool drought treatment (2016: 180 mm, 2017: 150 mm, 2018: 134 mm), and then ambient conditions (2016: 208 mm, 2017: 169 mm, 2018: 176 mm) (Figure 1b). Additionally, during much of the drought recovery period from June 2019 to April 2021, there was a long period of below average precipitation totals, coinciding with known severe regional drought conditions for that time period (Mankin et al. 2021).

**FIGURE 2:**
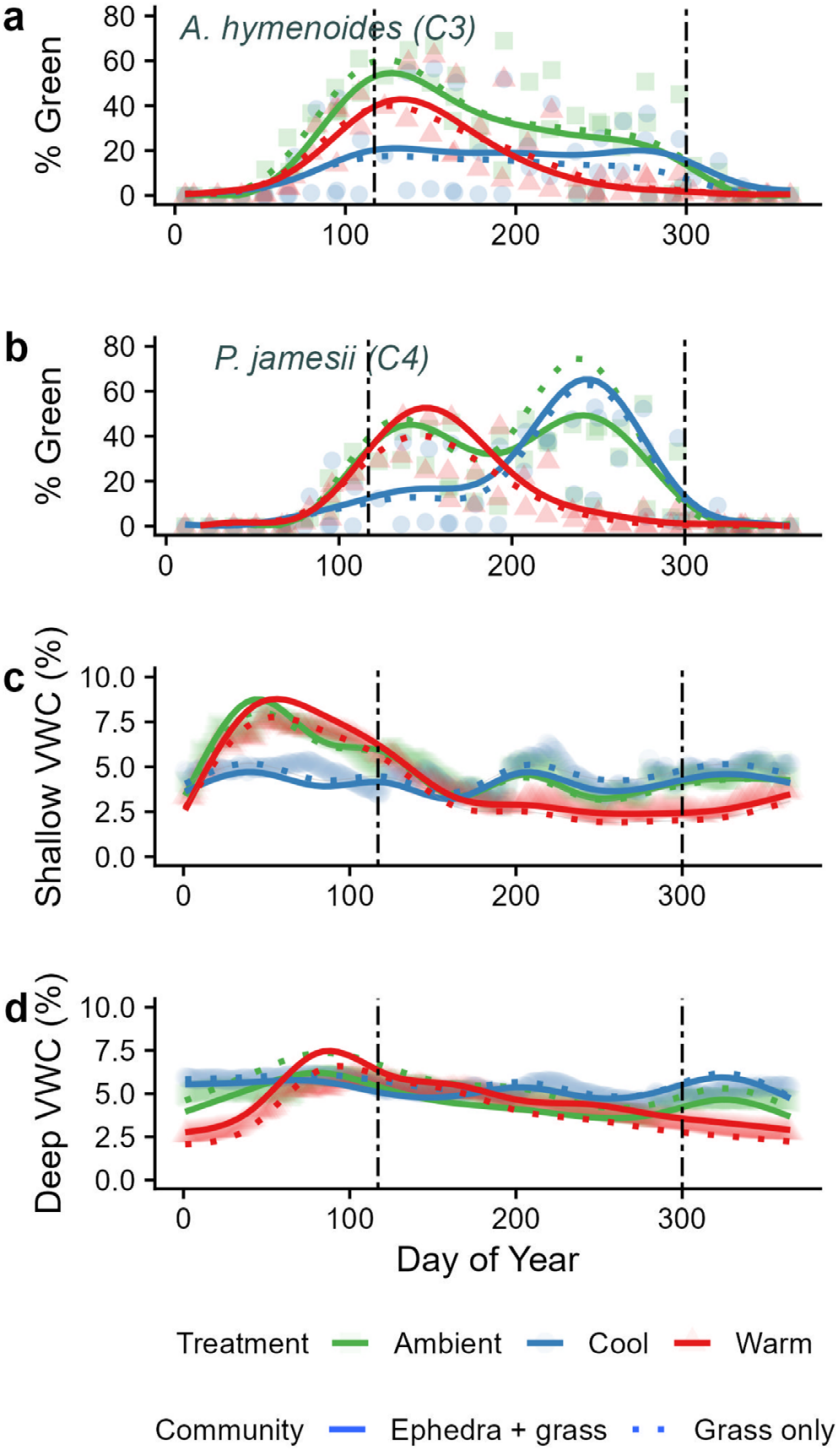
Generalized additive models for percent observed greenness (% Green) for *Achnatherum hymenoides* (a) and *Pleuraphis jamesii* (b), and shallow (5–25 cm) volumetric water content (VWC, %) (c), and deep (30–50 cm) volumetric water content (VWC, %) (d) by day of year across the drought experiment years (2016–2018). Generalized additive models for all variables used a smoothing term of drought treatment (ambient (green), cool (blue), and warm (red)) interacting plant community type (grass + *Ephedra viridis* (solid) or grass only (dash)). Associated statistics in Table S1. Dashed vertical lines indicate the DOY when rainout shelters were moved, with warm season rainout shelters in place ~DOY 117–300, and then moved for the cool‐season drought.

During the experimental drought, shallow (5–25 cm) volumetric water content (VWC) was noticeably higher in winter months (day of year ~1–120) in ambient and warm season drought conditions than in cool‐season drought conditions, while cool‐season drought treatments had less mean variance across the year (Figure 2c, Figure S1). Around day 175, mean shallow VWC was lowest in the warm season drought plots, with this trend continuing for the rest of the Julian year (Figure 2c). However, there were less noticeable treatment differences in the deep soil VWC (30–50 cm), especially during the growing season (~day of year 100–300). In the warm drought, mean deep soil VWC was lower in the winter months (Figure 2d), especially in the winter of 2015–2016 and 2018–2019. Following the removal of rainout shelters, treatment differences in soil moisture were minimized by June 15, 2019, approximately 50 days after shelter removal (Figure S1). However, due to severe ambient drought conditions from June 2019 to April 2021, there was little deep soil recharge and modest changes in deep soil VWC (Figure S1).

### 3.2 Phenological Responses of Target Grass Species

Both drought treatments impacted phenology and patterns of grass greenness for both *A. hymenoides* and *P. jamesii (*Figure 2a,b, Table S1, Adjusted *R* ^2^ = 0.47 for both species) with cool drought having the largest impact on *A. hymenoides* and warm season drought having the largest impact on *P. jamesii* estimated greenness (Figure 2a,b, Table S1). The presence of *E. viridis* had mixed effects, with increased total greenness for the two grass species, with generally negative impacts on mean greenness in ambient plots, and positive impacts for greenness in both drought treatments (Figure 2a,b). However, the plant community was not a very powerful predictor of total greenness when considering the total variation explained throughout the year (Figure 2a,b, Table S1). In *A. hymenoides* , mean peak greenness occurred around day 129 across treatments (Figure 2a). The pattern of mean greenness also varied by species and drought treatment. For ambient and warm drought conditions, modeled *A. hymenoides* largely had a unimodal pattern in greenness across the year. *P. jamesii* greenness had a bimodal pattern in ambient conditions (peak greenness occurring around DOY 140 and DOY 240), and a unimodal pattern, but in opposite seasons, for the two drought treatments (Figure 2a,b). *P. jamesii* greenness in the warm drought treatments peaked early (around day 146) while mean peak greenness in the cool‐season drought plots occurred around day 246 (Figure 2b). We also found that the rainout shelters were moved near the spring peak greenness for *A. hymenoides* , and toward the ends of the growing season for both grass species.

**FIGURE 3:**
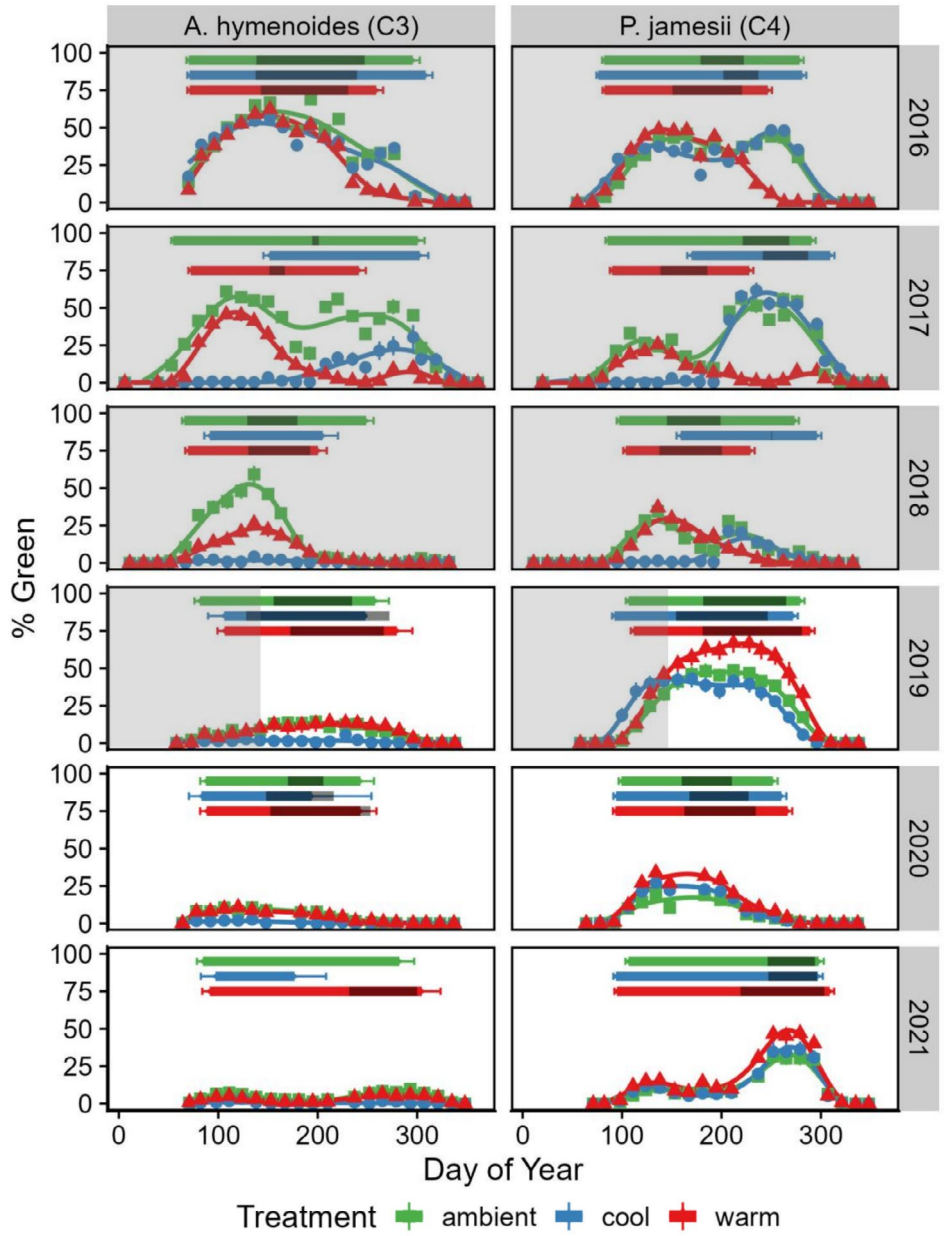
Mean grass greenness over time for the two dominant grass species, *A. hymenoides* (left side) and *P. jamesii* (right side). The bottom portion of each panel connects biweekly means (points) and error bars (±1 standard error) for each drought treatment (green squares = ambient, blue circle = cool, red triangle = warm). Bars in the upper portion of each panel indicate calculated phenological metrics, with the left side indicating mean start of growing season ±1 standard error, and the right side indicating the end of the growing season ±1 standard error. The shading on upper bars indicates the duration of developed inflorescences. Gray shading in the panels indicates the duration of the drought experiment (2016–2019) with final shelters removed 25 April 2019 (Day of Year = 115). Associated statistics are in Table S2.

Drought conditions impacted other phenological metrics, such as the start of the growing season (SOS), total growing season length, and end of the growing season (EGS), with the different grass species responding differently (Figure 3). In 2017, 2018, and 2019, the cool‐season drought treatment delayed the SOS for *A. hymenoides* (Figure 3) by 97, 25, and 70 days, respectively (Table S2). Cool‐season drought also delayed SOS for *P. jamesii* in 2017 and 2018 (Figure 3, Table S2) by 84 and 62 days, respectively, with a slight advancement in EGS by 14 days. Additionally, the timing of the SOS for mean ambient conditions *A. hymenoides* occurred 22.1 days earlier than the SOS for *P. jamesii* . However, in 2016, there were no observed differences in the SOS for either grass species by any drought treatment, which coincides with no observed treatment differences in volumetric soil moisture by early spring (~March 1, DOY 60). Warm season drought led to an earlier termination of the growing season for both species during the drought experiment from 2016 to 2018 (Figure 2a,b, Figure 3, Table S2) by 22–37 days. However, release from experimental drought conditions led to a slight increase in EGS for both grasses in 2019, when senescence delayed by 22 ( *A. hymenoides* ) and 10 days ( *P. jamesii* ). These constrictions on both the start and end of the growing season during the drought experiment often resulted in shorter growing seasons, most noticeably observed in 2017 and 2018 for both species and in both drought treatments (Figure 3, Table S2).

The timing of reproductive activity (i.e., inflorescence emergence) also shifted with drought, with a general trend of warm season drought shifting reproductive activity earlier in the year and cool‐season drought shifting reproductive activity later in the year for *P. jamesii* (Figure 3, Table S2). *A. hymenoides* reproductive timing was also sensitive to drought treatments, but this was most obviously observed through a lack of reproductive activities in the cool‐season drought plots in 2017 and 2018 (during the experiment) and in 2021 (after the experiment). In 2021, the ambient *A. hymenoides* also did not have any reproductive activity, while that same year we recorded some inflorescences only in the warm season drought plots (Figure 3, Table S2).

By 2021, following 2 years when all treatments received the same ambient precipitation, *P. jamesii* standardized greenness, and growing season metrics (start, end, and duration) showed little to no differences between ambient and drought treatment plots. Initially, when treatments ended in 2019, warm season drought plots showed a slight increase in the mean maximum greenness and growing season length (Table S2); but these treatment differences diminished in 2020 and 2021 (Figure 3). Mean maximum greenness was significantly lower in the 2020 growing season for all treatments but recovered to 2016 ambient values by 2021 (Figure 3, Table S2).

**FIGURE 4:**
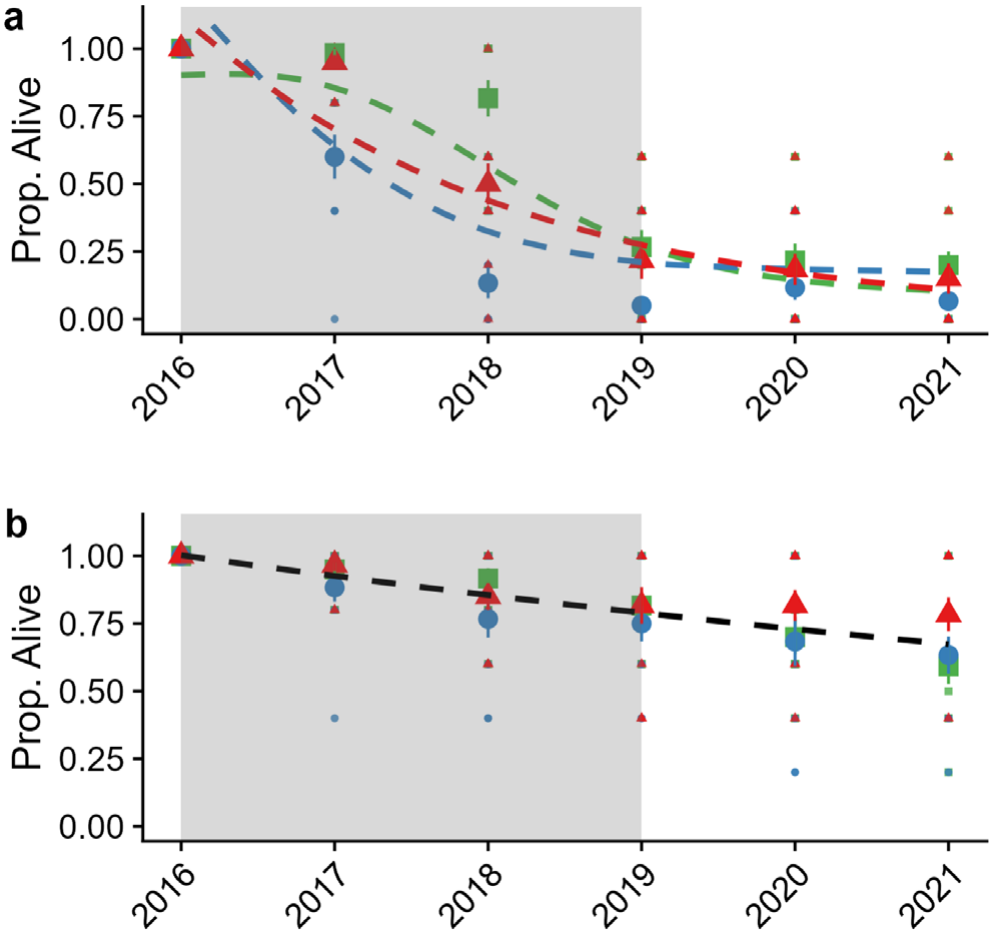
Models showing declines in the proportion of alive tagged individual (Prop. Alive) for *A. hymenoides* (a) and *P. jamesii* (b). For *A. hymenoides*, the probability of alive plants was predicted using a generalized additive model with a smoothing term of year by drought treatment (ambient = green, cool drought = blue, warm drought = red) with a binomial distribution. For *P. jamesii* , a binomial generalized linear model was used to predict changes in the proportion of alive plants by year with a binomial distribution. Larger symbols indicate mean values while smaller symbols represent plot level data. Gray shading box indicates years that the drought treatment was in place.

For *A. hymenoides* individuals, we observed declines in max. greenness starting in 2018 across treatments, which did not appear to be able to recover in 2019 or the years following the conclusion of the drought experiment (Figure 3, Table S2). Some of the large declines in mean max. greenness were likely related to high‐observed rates of mortality (Figure 4a, Table 2). For *A. hymenoides*, cool‐season drought appeared to have the most rapid mortality rates, with large declines in tagged individuals starting in 2017 (Figure 4a, Table 2). Ambient and warm season drought individuals also experienced high rates of mortality between 2018 and 2019, with only ~25% of tagged *A. hymenoides* surviving into 2021. However, *P. jamesii* mortality was not significantly impacted by drought treatment and was consistent throughout the study period (estimate probability of survival = −0.08 (±0.001), χ^2^ = 8.96, *p* = 0.002, Figure 4b, Table 2).

**TABLE 2:** Summary statistics for statistical models predicting mortality for *Achnatherum hymenoides* and *Pleuraphis jamesii* by drought treatment (ambient, cool drought, and warm drought) and year.

	Model type	Variable/Term	Estimate/EDF	*p*	*R* ^2^
*A. hymenoides*	*GAM*	(Intercept)	−0.76	< 0.001	0.72
		Cool drought	−0.90	< 0.001	
		Warm drought	−0.20	0.182	
		s(Year) × Ambient	2.37	< 0.001	
		s(Year) × Cool	2.71	< 0.001	
		s(Year) × Warm	1.00	< 0.001	
*P. jamesii*	*GLM*	(Intercept)	160.93	0.002	0.26
		Year	−0.08	0.002	
		Cool drought	−0.05	0.637	
		Warm drought	−0.05	0.617	

*Note:* For *A. hymenoides* , a generalized additive model (GAM) with treatment as a fixed effect and a smoothing term (denoted as s()) of year by drought treatment. For *P. jamesii* , the best model was a generalized linear model (GLM) with fixed effects of Year and drought treatments. Effective degrees of freedom (EDF) for the smoothing terms indicate the amount of curvature present, with large values representing greater curvature and values at or close to 1 indicating linearity.

## 4 Discussion

### 4.1 Phenological Responses Differ to Seasonal Drought

Our findings support the hypothesis that drought‐induced shifts in soil moisture availability affect grass species' phenology, which may be partially explained by different photosynthetic pathways. Notably, *P. jamesii* maintained or increased greenness in both the spring and late summer, even during hot conditions, if there was adequate summer precipitation. The observed bimodal greenness pattern in the C_4_ grass *P. jamesii* under ambient conditions, which transitioned to a unimodal response under drought treatments, highlights its phenological plasticity in response to altered soil moisture availability (Figure 2c). Similar shifts in green‐up timing of C_4_ grasses due to reduced moisture availability have been observed in other drylands (Rihan et al. 2022; Currier and Sala 2022), supporting the notion that warm season grasses may be able to alter seasonal growth when water is severely limited and await periods of higher soil moisture. Additionally, C_4_ dryland grasses may now also be benefiting from higher atmospheric concentrations of CO_2_ during drought conditions (Wang et al. 2022) which can help improve water‐use efficiency and maintain photosynthesis in increasingly hotter and drier climates (Del Toro et al. 2024). Therefore, the ability of C_4_ grasses to grow in the increasingly hotter, late‐summer periods following monsoon storms likely increases the drought resilience of this species.

Conversely, the C_3_ grass *A. hymenoides* showed less phenological flexibility, resistance, and resilience to both experimental and ambient drought conditions. The SOS for *A. hymenoides* occurred within a narrower range of dates in the spring (Table S2; except for cool drought in 2017), with little to no increase in greenness in late summer. This suggests that cool‐season C_3_ grasses may be the most vulnerable to depleted soil moisture conditions in the spring, and unable to compensate with later seasonal growth and/or a boost in productivity from increasing atmospheric CO_2_ (Figure 2a) (Wittmer et al. 2010; Ren et al. 2024). This aligns with the presumed reliance of C_3_ grasses on early‐season moisture for initiating growth (Winkler et al. 2020; Knapp et al. 2020). C_3_ grasses are likely better adapted to cooler temperatures and higher water availability (Winslow et al. 2003; Taylor et al. 2011) than the ambient and imposed climate conditions that occurred during this study (Comstock and Ehleringer 1992; Finger‐Higgens et al. 2023). For instance, during two of the four years of the experiment, ambient winter precipitation was below normal (Figure 1b), which might have limited *A. hymenoides* total growth and contributed to high rates of mortality across drought treatments (Figure 4a). Furthermore, the severe drought conditions that occurred during the recovery period of 2020–2021 may have further impeded recovery and resulted in large declines in *A. hymenoides* . We observed decreased cover in C_3_ grasses across all treatment plots, including ambient conditions, suggesting that climate conditions may already be limiting C_3_ grass growth in the greater region (Witwicki et al. 2016; Hoover et al. 2021; Finger‐Higgens et al. 2023).

Reproductive timing was also impacted by drought, with the driest treatments and years seeing little to no reproductive activity. In 2018, drought conditions appeared to be sufficiently severe to hinder any reproductive activity for either grass species in the cool drought treatments. Reproductive activity is often linked to water availability in grasses (Swemmer et al. 2006; Dietrich and Smith 2016), thus the 2018 spring moisture deficit may have been so severe as to limit production of reproductive parts in the later growing season. These results also suggest that spring moisture can be especially vital for C_3_ grasses, as this appears to be a critical time for both growth (i.e., greenness) and reproductive stimulation (Dietrich and Smith 2016; Hahn et al. 2021). As with phenology, C_4_ grasses demonstrated more flexibility in reproductive phenology, with reproduction activity occurring during periods of relatively higher soil moisture across experiment and recovery years. However, because this study only explored the presence of reproductive organs, we are unable to infer what the greater implications for drought might be on reproductive success and demographics of these two grass species.

Our study exploring relationships between seasonal drought, soil moisture, and phenology clarified the large impact that fall precipitation can have on soil moisture recharge in this ecosystem. Two of the four experiment years (2015, 2018) experienced large late October precipitation events before shelters were removed, which resulted in soil water recharge that lasted through the winter and provided available soil moisture for spring green‐up, while the warm season drought plots remained relatively dry (Figure S1). In both cases, the large storms occurred within the first 2 weeks of October, which is a period of low evapotranspiration (cool temperatures and after grasses senescence; Figure 3). Upon senescence, decreases in transpiration can be notable, which allows for water to accumulate in deeper soil layers (Schlaepfer et al. 2012). When this occurs in the fall, soils have yet to freeze, allowing for easier soil water percolation past shallower depths with high active root concentrations (Hoover et al. 2019). Traditionally, fall precipitation has not been considered important for dryland winter soil water recharge (Ehleringer et al. 1991), but these results reveal that autumnal storms following plant senescence can be vital to supporting ecosystems through periods of winter drought.

### 4.2 Mortality Dynamics and Drought Recovery

The hypothesis that *A. hymenoides* would have greater mortality under drought conditions compared to *P. jamesii* was largely supported. While both species experienced increased mortality under drought, the effect was more pronounced for the C_3_ species. This finding is consistent with other long‐term observational (Munson et al. 2011; Witwicki et al. 2016) and experimental studies (Winkler et al. 2019; Finger‐Higgens et al. 2023) from the region, which show consistent declines in *A. hymenoides* . Mortality may be due to the physiological trade‐offs associated with C_3_ photosynthesis, which prioritizes early growth and carbon assimilation under favorable conditions (Hoover et al. 2019) but is less efficient under water‐limited conditions (Taylor et al. 2011). When drought conditions become severe, C_3_ grasses may begin to experience increased mortality events, potentially due to hydraulic failure or carbon starvation (McDowell et al. 2008). Additionally, the shallow root profile of *A. hymenoides* may limit these plants from accessing deeper sources of soil moisture during extreme drought (Hoover et al. 2019), ultimately resulting in death.

By avoiding mortality events, C_4_ grasses showed greater resiliency to drought, with all treatment differences on phenology largely disappearing by the conclusion of this study (Figure 3). Additionally, because we did not observe any significant impacts of drought treatment on *P. jamesii* mortality rates (Figure 4b, Table 2), it does not appear that this experimental drought led to greater plant death than naturally occurring turnover and demographics. In fact, following the removal of the rainout shelters in 2019, we observed a slight increase in total greenness for *P. jamesii* in the warm season droughted plots, which may be due to compensatory growth following drought release (Zhou et al. 2022). Compensatory growth in grasses is hypothesized to be driven by soil dynamics deriving from drying and wetting cycles that can lead to increases in soil nitrogen (N) (Homyak et al. 2017; Schärer et al. 2023). In this experiment, we also observed increases in soil N in droughted plots at the end of the drought treatments in Fall 2018 and Spring 2019 (Finger‐Higgens, Hoover, et al. 2024), which may have supported a boost in *P. jamesii* growth in summer 2019. However, we did not see lasting impacts of drought on any of the phenological metrics for *P. jamesii* across treatments in 2020 and 2021, suggesting that drought legacies here may be transitory, and not lasting past one growing season.

### 4.3 Implications for Mixed C_3_ /C_4_ Grasslands

The observed declines in C_3_ grass from our study suggest that the composition of mixed C_3_/C_4_ grasslands is likely to continue to shift with more frequent and severe drought events. Historically across the CO Plateau, C_3_ and C_4_ grasses shared a mixed distribution, with both grasses covering 5%–30% of the area (Witwicki et al. 2016). However, over the last two decades there have been corroborating reports of declines in *A. hymenoides* grass cover, with high mortality rates, suggesting that climatic limitations to these C_3_ grasses may already be occurring (Munson et al. 2011; Hoover et al. 2015; Witwicki et al. 2016; Winkler et al. 2019; Finger‐Higgens et al. 2023). In some instances, C_4_ grass cover on the CO Plateau is predicted to either remain consistent or even slightly increase (Gremer et al. 2015; Havrilla et al. 2022), which could indicate shifting climatic envelopes for grasses with differing photosynthetic pathways. Given that many CO Plateau grasslands are managed for livestock (Copeland et al. 2017), predicted declines in *A. hymenoides* grasses will likely impact forage timing, quality, and quantity, because C_3_ grasses are often considered to be important spring forage because of their earlier spring green‐up and higher nutritional quality and palatability than C_4_ grasses (Barbehenn et al. 2004). Additionally, drought conditions may also decrease foliar nutrient quality for surviving grasses (León‐Sánchez et al. 2020), requiring grazing animals to consume more plant tissue to meet nutritional requirements. Management of livestock may need to adapt to changing species composition, both to provide sufficient nutrition for animals as well as to maintain grassland functional diversity by limiting grazing pressure on the few surviving C_3_ grasses. Native and domesticated grazers may then also become more reliant on the remaining C_4_ grasses, which could jeopardize grassland resiliency to warmer and drier conditions, and ultimately contribute to larger‐scale grass declines.

With declining grass populations, drought and aridification could also lead to more bare ground interspersed with a few hardy shrubs (Gremer et al. 2018). Previous research from this experiment supports this hypothesis, with experimental and ambient drought conditions leading to grass cover declines with corresponding increases in bare ground, while *E. viridis* biomass remained consistent (Finger‐Higgens, Hoover, et al. 2024). Additionally, grass species on the Colorado Plateau may also be vulnerable to increasing temperatures (Wertin et al. 2015), so combined increases in temperature and drought intensity and frequency may shift ecosystem composition. In many drylands, woody perennial species appear to be better adapted to increasing aridity compared to many dominant perennial grasses (Reynolds et al. 1999; Barger et al. 2011; Archer et al. 2017; Ren et al. 2024), bringing into question whether grass‐dominated (or even mixed grass‐shrub) drylands can persist in a warmer and drier world. Deeper‐rooted shrubs may fare better in extreme cases of drought, while more shallowly rooted perennial grasses and other herbaceous species may decline (Reynolds et al. 1999; Barger et al. 2011). These dynamics could threaten perennial grass stability, which could lead to the restructuring of grassland ecosystems toward more barren or shrubbier systems (Bestelmeyer et al. 2015; Archer et al. 2017; Bunting et al. 2017).

## 5 Conclusions

Overall, our findings underscore the divergent phenological and mortality responses of C_3_ and C_4_ grasses to drought conditions, emphasizing the vulnerability of *A. hymenoides* and the relative resilience of *P. jamesii* under changing drought regimes. The ability of *P. jamesii* to modify its growth and reproductive phenology to periods of increased moisture, even under severe drought, highlights the potential for C_4_ grasses to persist and possibly expand in warming, arid environments. In contrast, the restricted phenological flexibility and high mortality rates of *A. hymenoides* point to significant challenges for C_3_ grasses in these ecosystems. The broader implications for mixed C_3_/C_4_ grasslands include shifts in species composition, reduced forage availability, and increased susceptibility to ecosystem restructuring toward woody vegetation or degraded landscapes. As drought events become more frequent and intense, these findings provide critical insights into the mechanisms driving grassland resilience and inform future management strategies for maintaining grassland function and biodiversity in the face of climate change.

## Author Contributions

**Rebecca A. Finger Higgens:** formal analysis (lead), investigation (supporting), visualization (lead), writing – original draft (lead). **David L. Hoover:** conceptualization (equal), formal analysis (supporting), investigation (lead), methodology (lead), writing – review and editing (equal). **Anna C. Knight:** data curation (equal), formal analysis (supporting), methodology (equal), visualization (supporting), writing – review and editing (equal). **Daniel R. Schlaepfer:** methodology (supporting), software (supporting), writing – review and editing (equal). **Michael C. Duniway:** conceptualization (equal), formal analysis (supporting), funding acquisition (lead), investigation (equal), project administration (lead), supervision (lead), writing – review and editing (equal).

## Conflicts of Interest

The authors declare no conflicts of interest.

## Supporting information


Data S1.

## Data Availability

Data are available at Finger‐Higgens et al. (2025), https://doi.org/10.5066/P13YQBFB.
