# Supplementary material for: Flexible Phenology of a C4 Grass Linked to Resiliency to Seasonal and Multiyear Drought Events in the American Southwest
Source: Ecol Evol. 2025 May 14;15(5):e71435. doi: 10.1002/ece3.71435 (PMC12077930; doi:10.1002/ece3.71435)
Supplement: Supplementary file 1 — Data S1. [file ECE3-15-e71435-s001.docx]

*Supplemental material for:*

**Flexible phenology of a C_4_ grass linked to resiliency to seasonal and multiyear drought events in the American Southwest**

Rebecca A. Finger Higgens^1^, David L. Hoover^2^, Anna C. Knight^1^, Daniel R. Schlaepfer^3,4^, Michael C. Duniway^1^

^1^ US Geological Survey, Southwest Biological Science Center, Moab, UT, USA

^2^ USDA-ARS Rangeland Resources and Systems Research Unit, Fort Collins, CO, USA

^3^ US Geological Survey, Southwest Biological Science Center, Flagstaff, AZ, USA

^4^ Center for Adaptable Western Landscapes, Northern Arizona University, Flagstaff, AZ, USA

*contact/corresponding author

Email: rfinger-higgens@usgs.gov

The following Supporting Information is available for this article:

**Fig. S1:** Timeseries of soil volumetric water content at shallow (5-25 cm) and deep (30-50 cm) depths and between different drought treatments from 2015-2021.

**Table S1:** Summary statistics from generalized additive models predicting seasonal patterns of *Achanatherum hymenoides* and *Pleuraphis jamesii* greenness and shallow (5-25 cm) and deep (30-50 cm) soil volumetric water content (VWC %) by drought treatments (ambient, cool drought, warm drought) and plant community (grass only or grass + *Ephedra*) during the active drought period of 2016-2018

**Table S2:** Estimated marginal means for linear mixed effects models of phenological metrics for each year from 2016-2021 for *Achanatherum hymenoides* and *Pleuraphis jamesii*.


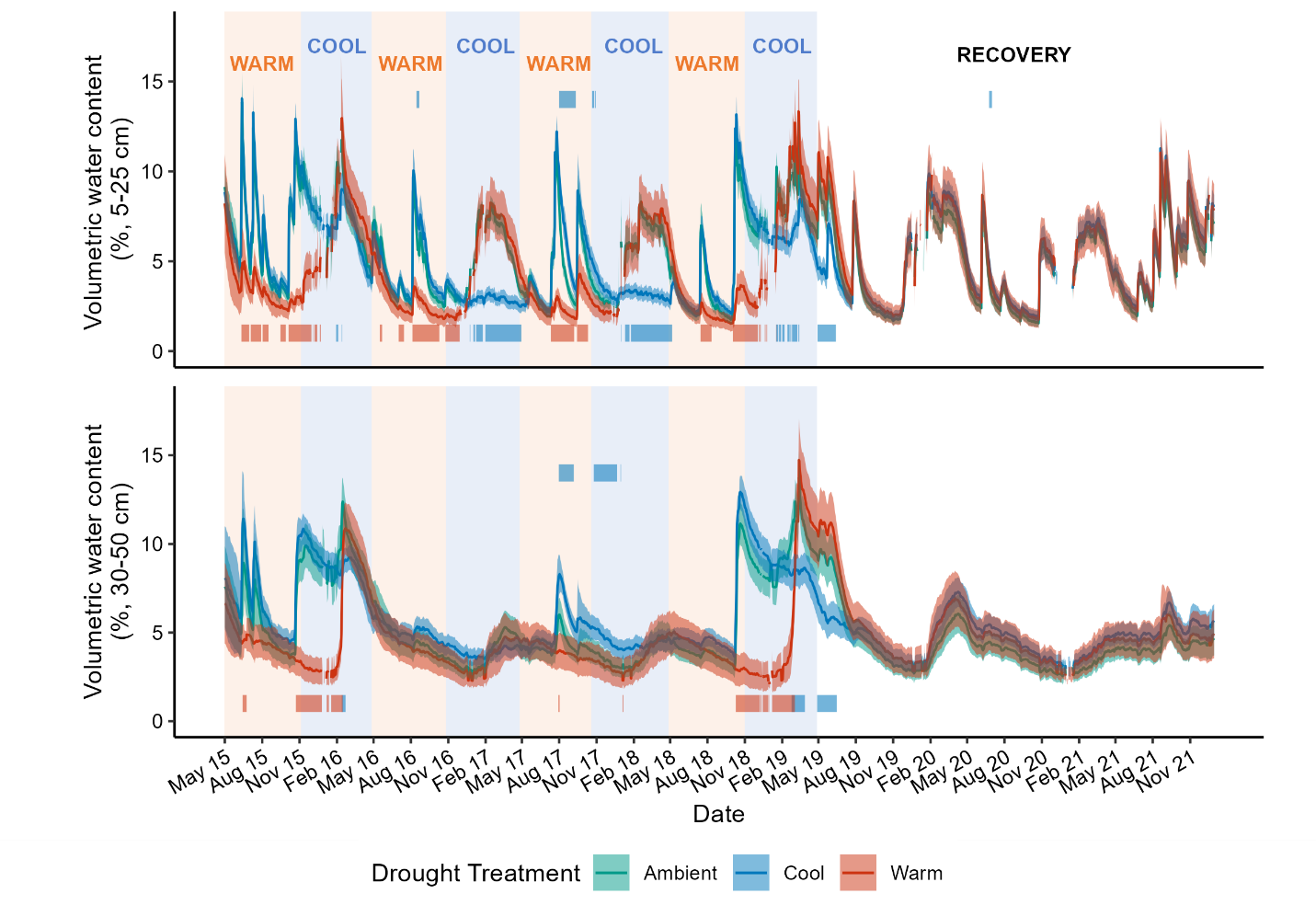


**Fig. S1** Timeseries of soil volumetric water content (%) at shallow (5-25 cm) and deep (30-50 cm) depths and between different drought treatments (ambient, cool, and warm) from 2015-2021. Vertical shading bars indicate when seasonal drought treatments were present. Dark red and blue bars above and below soil moisture indicate treatments are greater than ambient (positive effect), while bars below indicate that they are less than ambient (negative effects). Data were removed when soil temperatures were at or below freezing.

**Table S1:** Summary statistics from generalized additive models predicting seasonal patterns of *Achanatherum hymenoides* and *Pleuraphis jamesii* greenness and shallow (5-25 cm) and deep (30-50 cm) soil volumetric water content (VWC %) by drought treatments (ambient, cool drought, warm drought) and plant community (grass only or grass + *Ephedra*) during the active drought period of 2016-2018. Presented statistics include parametric coefficients estimates, 95 % confidence intervals (CI), and p-values, and smoothing terms effective degrees of freedom (edf), and p-values. R^2^ represented the portion of variance explained by the total model. The smoothing term of s(PlotID) represents the random effects to account for repeat measures and non-independence of observations.

|  | Parametric coefficient | Estimate | *CI* | p-value |  | Smoothing Term | edf | p-value | R^2^ |
| --- | --- | --- | --- | --- | --- | --- | --- | --- | --- |
| *A. HYMENODIES* | (Intercept) | 28.08 | 26.48 – 29.68 | <0.001 |  | s(DOY):Ambient.Ephedra + grass | 8.1 | <0.001 | 0.47 |
|  | Cool drought | -17.27 | -19.52 – -15.03 | <0.001 |  | s(DOY):Cool drought.Ephedra + grass | 3.2 | <0.001 |  |
|  | Warm drought | -12.45 | -14.76 – -10.13 | <0.001 |  | s(DOY):Warm drought.Ephedra + grass | 7.4 | <0.001 |  |
|  | Grass Only | -3.07 | -5.29 – -0.85 | 0.007 |  | s(DOY):Ambient.Grass only | 7.8 | <0.001 |  |
|  | Cool drought × Grass Only | 6.4 | 3.26 – 9.54 | <0.001 |  | s(DOY):Cool drought.Grass only | 5.6 | <0.001 |  |
|  | Warm drought × Grass Only | 2.8 | -0.34 – 5.94 | 0.081 |  | s(DOY):Warm drought.Grass only | 7.1 | <0.001 |  |
|  |  |  |  |  |  | s(PlotID) | 0.9 | 0.009 |  |
|  |  |  |  |  |  |  |  |  |  |
| *P. jameseii* | (Intercept) | 22.16 | 20.39 – 23.94 | <0.001 |  | s(DOY):Ambient.Ephedra + grass | 8.5 | <0.001 | 0.47 |
|  | Cool drought | -6.37 | -8.36 – -4.37 | <0.001 |  | s(DOY):Cool drought.Ephedra + grass | 7.8 | <0.001 |  |
|  | Warm drought | -10.39 | -12.39 – -8.39 | <0.001 |  | s(DOY):Warm drought.Ephedra + grass | 7.7 | <0.001 |  |
|  | Grass Only | -2.28 | -4.28 – -0.28 | 0.025 |  | s(DOY):Ambient.Grass only | 8.1 | <0.001 |  |
|  | Cool drought × Grass Only | 3.73 | 0.91 – 6.56 | 0.01 |  | s(DOY):Cool drought.Grass only | 7.7 | <0.001 |  |
|  | Warm drought × Grass Only | 4.33 | 1.51 – 7.16 | 0.003 |  | s(DOY):Warm drought.Grass only | 8.0 | <0.001 |  |
|  |  |  |  |  |  | s(PlotID) | 0.8 | <0.001 |  |
|  |  |  |  |  |  |  |  |  |  |
| Shallow VWC % | (Intercept) | 5.74 | 5.68 – 5.80 | <0.001 |  | s(DOY):Ambient.Ephedra + grass | 9.0 | <0.001 | 0.32 |
|  | Cool drought | -0.7 | -0.77 – -0.64 | <0.001 |  | s(DOY):Cool drought.Ephedra + grass | 9.0 | <0.001 |  |
|  | Warm drought | -0.9 | -0.96 – -0.84 | <0.001 |  | s(DOY):Warm drought.Ephedra + grass | 8.92 | <0.001 |  |
|  | Grass Only | -0.18 | -0.25 – -0.12 | <0.001 |  | s(DOY):Ambient.Grass only | 8.98 | <0.001 |  |
|  | Cool drought × Grass Only | 0.65 | 0.56 – 0.74 | <0.001 |  | s(DOY):Cool drought.Grass only | 8.97 | <0.001 |  |
|  | Warm drought × Grass Only | 0.11 | 0.02 – 0.20 | 0.018 |  | s(DOY):Warm drought.Grass only | 8.93 | <0.001 |  |
|  |  |  |  |  |  | s(PlotID) | 1.00 | <0.001 |  |
|  |  |  |  |  |  |  |  |  |  |
| Deep VWC % | (Intercept) | 5.69 | 5.63 – 5.75 | <0.001 |  | s(DOY):Ambient.Ephedra + grass | 8.72 | <0.001 | 0.24 |
|  | Cool drought | 0.6 | 0.53 – 0.66 | <0.001 |  | s(DOY):Cool drought.Ephedra + grass | 8.74 | <0.001 |  |
|  | Warm drought | 0.03 | -0.04 – 0.10 | 0.36 |  | s(DOY):Warm drought.Ephedra + grass | 8.97 | <0.001 |  |
|  | Grass Only | 0.88 | 0.81 – 0.94 | <0.001 |  | s(DOY):Ambient.Grass only | 8.72 | <0.001 |  |
|  | Cool drought × Grass Only | -0.55 | -0.65 – -0.46 | <0.001 |  | s(DOY):Cool drought.Grass only | 8.80 | <0.001 |  |
|  | Warm drought × Grass Only | -1.82 | -1.92 – -1.73 | <0.001 |  | s(DOY):Warm drought.Grass only | 8.95 | <0.001 |  |
|  |  |  |  |  |  | s(PlotID) | 1.00 | <0.001 |  |

**Table S2:** Estimated marginal means (EMM) for linear mixed effects models of phenological metrics for each year from 2016-2021 for *Achanatherum hymenoides* and *Pleuraphis jamesii*. Phenological metrics include: start of growing season, end of growing season, growing season length, start of reproductive activity, end of reproductive activity, and reproductive activity length. Drought treatments (ambient, cool drought, and warm drought) were active from 2016- Spring 2019, with 2020 and 2021 measurements occurring during experimental drought recovery. Standard error (SE) and denominator degrees of freedom (df) are report, as are sidak post-hoc multiple comparisons where different letters indicate differences in phenological metrics by year and treatment for a given species.

|  | *A. hymenoids* | |  |  |  |  |  | *P. jamesii* | |  |  |  |  |
| --- | --- | --- | --- | --- | --- | --- | --- | --- | --- | --- | --- | --- | --- |
|  | **Year** | **Treatment** | **EMM** | **SE** | **df** | **PostHoc** |  | **Year** | **Treatment** | **EMM** | **SE** | **df** | **PostHoc** |
| Start of Growing Season | 2016 | ambient | 70.3 | 2.3 | 41.7 | de |  | 2016 | ambient | 81.9 | 2.0 | 29.2 | hi |
|  | 2016 | cool | 71.2 | 2.3 | 40.8 | de |  | 2016 | cool | 76.1 | 1.8 | 28.6 | i |
|  | 2016 | warm | 71.2 | 2.3 | 40.8 | de |  | 2016 | warm | 82.3 | 2.0 | 28.6 | hi |
|  | 2017 | ambient | 54.5 | 1.8 | 41.7 | f |  | 2017 | ambient | 85.4 | 2.1 | 30.4 | gh |
|  | 2017 | cool | 151.4 | 6.1 | 84.6 | a |  | 2017 | cool | 169.8 | 4.2 | 34.9 | a |
|  | 2017 | warm | 72.3 | 2.4 | 43.7 | cde |  | 2017 | warm | 89.9 | 2.2 | 29.8 | fgh |
|  | 2018 | ambient | 65.9 | 2.3 | 53.5 | e |  | 2018 | ambient | 97.1 | 2.4 | 31.8 | cdef |
|  | 2018 | cool | 91.4 | 5.5 | 228.8 | bc |  | 2018 | cool | 158.9 | 4.1 | 39.9 | a |
|  | 2018 | warm | 69.6 | 2.9 | 95.2 | de |  | 2018 | warm | 103.7 | 2.6 | 33.2 | bcde |
|  | 2019 | ambient | 81.4 | 5.4 | 306.9 | bcde |  | 2019 | ambient | 106.5 | 2.7 | 38.6 | bc |
|  | 2019 | cool | 105.9 | 16.1 | 428.4 | abcde |  | 2019 | cool | 92.4 | 2.5 | 44.8 | fg |
|  | 2019 | warm | 106.3 | 7.1 | 295.9 | b |  | 2019 | warm | 111.7 | 2.8 | 37.6 | b |
|  | 2020 | ambient | 87.4 | 5.6 | 285.5 | bcd |  | 2020 | ambient | 99.3 | 2.7 | 46 | cdef |
|  | 2020 | cool | 83 | 12.5 | 455.7 | bcdef |  | 2020 | cool | 93.9 | 2.6 | 51.2 | efg |
|  | 2020 | warm | 88.2 | 6.5 | 330 | bcd |  | 2020 | warm | 93.3 | 2.4 | 38.6 | fg |
|  | 2021 | ambient | 84.4 | 5.9 | 316.2 | bcde |  | 2021 | ambient | 106.4 | 3.0 | 54.7 | bcd |
|  | 2021 | cool | 97.1 | 14.6 | 449.2 | abcde |  | 2021 | cool | 93.8 | 2.6 | 49.4 | efg |
|  | 2021 | warm | 91.4 | 7.5 | 370.4 | bcd |  | 2021 | warm | 94.7 | 2.4 | 38.6 | defg |
|  |  |  |  |  |  |  |  |  |  |  |  |  |  |
| End of Growing Season | 2016 | ambient | 296 | 6.9 | 58.1 | ab |  | 2016 | ambient | 279 | 4.1 | 93.8 | bcde |
|  | 2016 | cool | 309 | 6.9 | 56.7 | a |  | 2016 | cool | 281 | 4.0 | 91.3 | bcd |
|  | 2016 | warm | 259 | 6.9 | 56.7 | c |  | 2016 | warm | 247 | 4.0 | 91.3 | gh |
|  | 2017 | ambient | 301 | 6.9 | 58.1 | ab |  | 2017 | ambient | 291 | 4.1 | 99 | abc |
|  | 2017 | cool | 303 | 8.7 | 121.1 | a |  | 2017 | cool | 309 | 4.3 | 117.5 | a |
|  | 2017 | warm | 241 | 7.0 | 60.8 | cd |  | 2017 | warm | 228 | 4.1 | 96.3 | i |
|  | 2018 | ambient | 249 | 7.4 | 74.8 | cd |  | 2018 | ambient | 274 | 4.2 | 104.5 | cde |
|  | 2018 | cool | 205 | 15.3 | 341.5 | de |  | 2018 | cool | 296 | 4.5 | 136.4 | ab |
|  | 2018 | warm | 200 | 8.8 | 127.5 | e |  | 2018 | warm | 229 | 4.2 | 110.5 | hi |
|  | 2019 | ambient | 258 | 14.0 | 328.1 | abcd |  | 2019 | ambient | 279 | 4.5 | 132.9 | bcde |
|  | 2019 | cool | 256 | 32.4 | 401.6 | abcde |  | 2019 | cool | 272 | 4.7 | 156.1 | cdef |
|  | 2019 | warm | 280 | 15.6 | 347.1 | abc |  | 2019 | warm | 289 | 4.5 | 129.4 | abc |
|  | 2020 | ambient | 243 | 13.6 | 313.4 | cde |  | 2020 | ambient | 252 | 4.8 | 163.1 | fg |
|  | 2020 | cool | 190 | 32.3 | 429.8 | bcde |  | 2020 | cool | 260 | 4.9 | 178.6 | efg |
|  | 2020 | warm | 243 | 16.2 | 352.7 | abcde |  | 2020 | warm | 267 | 4.5 | 134.5 | def |
|  | 2021 | ambient | 282 | 14.8 | 330.8 | abc |  | 2021 | ambient | 298 | 5.0 | 197.1 | ab |
|  | 2021 | cool | 177 | 31.7 | 398.9 | cde |  | 2021 | cool | 297 | 4.9 | 174.7 | ab |
|  | 2021 | warm | 305 | 18.9 | 379.7 | abc |  | 2021 | warm | 309 | 4.5 | 137.3 | a |
|  |  |  |  |  |  |  |  |  |  |  |  |  |  |
| Growing Season length | 2016 | ambient | 225.4 | 8.1 | 44.7 | ab |  | 2016 | ambient | 196 | 5.7 | 27.6 | ab |
|  | 2016 | cool | 236.8 | 8.0 | 43.6 | a |  | 2016 | cool | 205 | 5.7 | 27.1 | a |
|  | 2016 | warm | 187.6 | 8.0 | 43.6 | bc |  | 2016 | warm | 164 | 5.7 | 27.1 | cd |
|  | 2017 | ambient | 245.7 | 8.1 | 44.7 | a |  | 2017 | ambient | 204 | 5.8 | 28.8 | a |
|  | 2017 | cool | 148.1 | 9.8 | 89.2 | cde |  | 2017 | cool | 137 | 6.0 | 33.2 | fg |
|  | 2017 | warm | 167.9 | 8.2 | 46.7 | c |  | 2017 | warm | 137 | 5.8 | 28.2 | efg |
|  | 2018 | ambient | 179.8 | 8.6 | 56.5 | c |  | 2018 | ambient | 176 | 5.9 | 30.1 | bc |
|  | 2018 | cool | 103.6 | 14.5 | 230.1 | e |  | 2018 | cool | 131 | 6.2 | 37.9 | fg |
|  | 2018 | warm | 123.9 | 10.1 | 98 | de |  | 2018 | warm | 123 | 5.9 | 31.4 | g |
|  | 2019 | ambient | 172.6 | 15.7 | 290.6 | bcde |  | 2019 | ambient | 170 | 6.2 | 36.6 | cd |
|  | 2019 | cool | 145.1 | 35.7 | 383.9 | abcde |  | 2019 | cool | 177 | 6.4 | 42.6 | bcd |
|  | 2019 | warm | 166.5 | 15.9 | 285.2 | bcde |  | 2019 | warm | 175 | 6.1 | 35.6 | bcd |
|  | 2020 | ambient | 145.2 | 15.2 | 273 | cde |  | 2020 | ambient | 150 | 6.4 | 43.6 | def |
|  | 2020 | cool | 95.5 | 35.8 | 409.8 | cde |  | 2020 | cool | 162 | 6.6 | 48.5 | cde |
|  | 2020 | warm | 154.7 | 17.4 | 313.2 | cde |  | 2020 | warm | 171 | 6.2 | 36.5 | cd |
|  | 2021 | ambient | 192.1 | 16.4 | 299.5 | abc |  | 2021 | ambient | 185 | 6.7 | 51.6 | abc |
|  | 2021 | cool | 67.4 | 35.5 | 396.4 | cde |  | 2021 | cool | 201 | 6.6 | 46.9 | ab |
|  | 2021 | warm | 192.4 | 19.4 | 343.2 | abcd |  | 2021 | warm | 210 | 6.2 | 36.5 | a |
|  |  |  |  |  |  |  |  |  |  |  |  |  |  |
| Start of Reproductive Activity | 2016 | ambient | 138 | 2.5 | 41.9 | a |  | 2016 | ambient | 178.95 | 7.5 | 169.9 | abcd |
|  | 2016 | cool | 137 | 2.9 | 61.7 | ab |  | 2016 | cool | 201.84 | 8.8 | 237.6 | cde |
|  | 2016 | warm | 143 | 2.5 | 42.9 | bc |  | 2016 | warm | 150.64 | 8.0 | 198.4 | ab |
|  | 2017 | ambient | 194 | 7.8 | 173 | de |  | 2017 | ambient | 221.36 | 8.3 | 213.7 | efa |
|  | 2017 | cool | NA | NA | NA |  |  | 2017 | cool | 241.51 | 7.1 | 131.0 | fa |
|  | 2017 | warm | 151 | 7.8 | 173.1 | abc |  | 2017 | warm | 138.67 | 17.9 | 399.6 | abc |
|  | 2018 | ambient | 129 | 3.0 | 63.1 | a |  | 2018 | ambient | 145.08 | 8.7 | 227.6 | ab |
|  | 2018 | cool | NA | NA | NA |  |  | 2018 | cool | 250.74 | 25.2 | 398.8 | defa |
|  | 2018 | warm | 130 | 4.2 | 133.1 | a |  | 2018 | warm | 137.56 | 9.7 | 293.6 | a |
|  | 2019 | ambient | 156 | 8.7 | 164.7 | abcd |  | 2019 | ambient | 181.59 | 7.7 | 185.0 | bcd |
|  | 2019 | cool | 128 | 17.4 | 190.8 | abc |  | 2019 | cool | 154.33 | 8.0 | 190.4 | ab |
|  | 2019 | warm | 172 | 6.7 | 149.6 | cde |  | 2019 | warm | 181.02 | 6.6 | 123.5 | bcd |
|  | 2020 | ambient | 170 | 8.7 | 154.3 | bcde |  | 2020 | ambient | 159.94 | 10.3 | 312.4 | abcd |
|  | 2020 | cool | 148 | 17.4 | 190.8 | abcde |  | 2020 | cool | 167.77 | 10.1 | 288.6 | abcd |
|  | 2020 | warm | 152 | 8.8 | 139.6 | abc |  | 2020 | warm | 162.65 | 6.8 | 134.6 | abcd |
|  | 2021 | ambient | NA | NA | NA |  |  | 2021 | ambient | 246.39 | 7.3 | 169.9 | fa |
|  | 2021 | cool | NA | NA | NA |  |  | 2021 | cool | 247.15 | 7.3 | 136.6 | fa |
|  | 2021 | warm | 232 | 17.3 | 148.9 | e |  | 2021 | warm | 219.11 | 6.4 | 112.5 | efa |
|  |  |  |  |  |  |  |  |  |  |  |  |  |  |
| End of Reproductive Period | 2016 | ambient | 247.1 | 5.0 | 20.5 | de |  | 2016 | ambient | 222.6 | 5.5 | 260.2 | abc |
|  | 2016 | cool | 239.5 | 5.5 | 28.4 | cde |  | 2016 | cool | 237.1 | 6.7 | 306.4 | bcd |
|  | 2016 | warm | 230.7 | 5.0 | 21.0 | cd |  | 2016 | warm | 220.71 | 6.0 | 276.2 | abc |
|  | 2017 | ambient | 201.1 | 12.0 | 148.2 | abc |  | 2017 | ambient | 268.18 | 6.3 | 277.8 | defa |
|  | 2017 | cool | NA | NA | NA |  |  | 2017 | cool | 287.07 | 5.1 | 214.8 | efafb |
|  | 2017 | warm | 166.9 | 12.0 | 148.2 | a |  | 2017 | warm | 185.8 | 14.4 | 399.9 | ab |
|  | 2018 | ambient | 179.6 | 5.6 | 30.5 | a |  | 2018 | ambient | 199.1 | 6.6 | 280.7 | a |
|  | 2018 | cool | NA | NA | NA |  |  | 2018 | cool | 250.0 | 20.4 | 400.0 | abcdef |
|  | 2018 | warm | 192.4 | 7.1 | 66.3 | ab |  | 2018 | warm | 200.40 | 7.5 | 353.7 | a |
|  | 2019 | ambient | 234.5 | 13.3 | 143.5 | bcde |  | 2019 | ambient | 265.01 | 5.8 | 275.6 | de |
|  | 2019 | cool | 272.1 | 26.6 | 176.9 | bcde |  | 2019 | cool | 246.52 | 6.0 | 282.7 | cd |
|  | 2019 | warm | 266.5 | 10.5 | 127.0 | e |  | 2019 | warm | 280.89 | 4.8 | 207.7 | efghi |
|  | 2020 | ambient | 205.7 | 13.4 | 133.0 | abcd |  | 2020 | ambient | 210.45 | 8.1 | 368.1 | ab |
|  | 2020 | cool | 216.1 | 26.6 | 176.9 | abcde |  | 2020 | cool | 227.34 | 7.8 | 347.3 | abc |
|  | 2020 | warm | 253.0 | 13.5 | 123.1 | cde |  | 2020 | warm | 234.40 | 5.0 | 213.1 | bc |
|  | 2021 | ambient | NA | NA | NA |  |  | 2021 | ambient | 293.95 | 5.4 | 271.7 | fghi |
|  | 2021 | cool | NA | NA | NA |  |  | 2021 | cool | 296.15 | 5.3 | 218.1 | fghi |
|  | 2021 | warm | 299.9 | 25.6 | 126.6 | de |  | 2021 | warm | 303.43 | 4.6 | 189.2 | fghi |
|  |  |  |  |  |  |  |  |  |  |  |  |  |  |
| Reproductive Activity Length | 2016 | ambient | 86.9 | 5.9 | 59.7 | a |  | 2016 | ambient | 20.44 | 5.9 | 99.9 | e ghij |
|  | 2016 | cool | 59.7 | 5.8 | 58.3 | abc |  | 2016 | cool | 11.88 | 5.9 | 97.8 | ghij |
|  | 2016 | warm | 67.7 | 5.8 | 58.3 | ab |  | 2016 | warm | 28.50 | 5.9 | 97.8 | cdefg i |
|  | 2017 | ambient | 0.7 | 5.9 | 59.7 | gh |  | 2017 | ambient | 18.12 | 6.0 | 104.7 | ghij |
|  | 2017 | cool | 1.6 | 7.2 | 115.6 | efgh |  | 2017 | cool | 30.20 | 6.3 | 121.0 | cdefgh |
|  | 2017 | warm | 1.9 | 5.9 | 62.3 | f h |  | 2017 | warm | 2.96 | 6.0 | 102.2 | h j |
|  | 2018 | ambient | 33.7 | 6.3 | 75.3 | cdef |  | 2018 | ambient | 19.92 | 6.1 | 109.8 | e ghij |
|  | 2018 | cool | 3.8 | 10.7 | 266.9 | defgh |  | 2018 | cool | -1.54 | 6.5 | 138.4 | ij |
|  | 2018 | warm | 32.6 | 7.4 | 128.1 | cdefg |  | 2018 | warm | 17.36 | 6.2 | 115.0 | fghij |
|  | 2019 | ambient | 22.9 | 11.6 | 339.4 | bcdefgh |  | 2019 | ambient | 46.32 | 6.5 | 134.9 | abcdef |
|  | 2019 | cool | 73.1 | 26.5 | 402.2 | abcdefgh |  | 2019 | cool | 52.95 | 6.8 | 156.1 | abcde |
|  | 2019 | warm | 59.9 | 11.7 | 327.7 | abcd |  | 2019 | warm | 77.94 | 6.4 | 130.9 | a |
|  | 2020 | ambient | 6.2 | 11.2 | 321.4 | defgh |  | 2020 | ambient | 15.93 | 6.8 | 161.6 | ghij |
|  | 2020 | cool | 24.5 | 26.4 | 431.4 | abcdefgh |  | 2020 | cool | 20.70 | 7.0 | 176.7 | d fghij |
|  | 2020 | warm | 46.1 | 12.8 | 351.1 | abcdefgh |  | 2020 | warm | 53.57 | 6.5 | 134.5 | abcd |
|  | 2021 | ambient | -7.7 | 12.1 | 342.6 | efgh |  | 2021 | ambient | 37.44 | 7.2 | 191.4 | bcdefg |
|  | 2021 | cool | -9.5 | 26.3 | 419.3 | abcdefgh |  | 2021 | cool | 37.85 | 7.0 | 171.2 | bcdefg |
|  | 2021 | warm | -10.4 | 14.3 | 376.7 | efgh |  | 2021 | warm | 72.47 | 6.5 | 134.8 | ab |
